# Gradual increases in sugar concentration enhance total perceived sweetness in individuals prone to sweetness habituation

**DOI:** 10.3389/fpsyg.2025.1561107

**Published:** 2025-07-15

**Authors:** Ryota Nitto, Yuki Ban, Rui Fukui, Shin'ichi Warisawa

**Affiliations:** Department of Human and Engineered Environmental Studies, Graduate School of Frontier Sciences, The University of Tokyo, Kashiwa, Chiba, Japan

**Keywords:** sweetness, perception, habituation, contrast effect, sensory adaptation

## Abstract

**Introduction:**

Excessive sugar consumption has become a major health concern, contributing to obesity and diabetes. To address this issue without increasing sugar intake, various strategies to enhance sweetness have been explored. However, conventional methods, which rely on repetitive stimuli or patterns, fail to counteract the decline in perceived sweetness caused by habituation during continuous consumption.

**Methods:**

We hypothesized that gradually increasing sugar concentration during consumption could mitigate habituation and enhance overall sweetness perception. To test this hypothesis, we developed a system capable of delivering sucrose solutions with gradually changing concentrations. In the experiment, participants consumed a 15-second continuous flow of sucrose solution with an average concentration of 4.7%. The sugar concentration either increased from 3.8% to 5.6%, decreased from 5.6% to 3.8%, or remained constant. Participants were instructed to swallow at 3-second intervals for a total of five times, rating sweetness after each swallow. The total perceived sweetness, measured as the Area Under the Curve (AUC), was compared across conditions.

**Results:**

In groups showing habituation under constant concentration, the increasing concentration condition improved total perceived sweetness.

**Discussion:**

These findings suggest that increasing sugar concentration patterns can alleviate habituation and enhance sweetness perception, particularly in individuals prone to sweetness habituation.

## 1 Introduction

Food experiences, including beverages, are essential not only for sustaining life but also for enhancing social, cultural, and psychological aspects of quality of life (Barr and Schumacher, 2003). In particular, sweetness imparted by food is regarded as an innately preferred taste quality for humans (Divert et al., 2017) and is indispensable for the enjoyment of food experiences. For instance, sugar is sometimes added to beverages with acquired tastes, such as tea or coffee, to make them more palatable. Moreover, sweetened beverages in plastic bottles are consumed daily across the globe. In this way, sweetened beverages and other foods containing sweetness contribute significantly to the pleasure of eating.

However, sweeteners commonly used in food products pose significant health risks, necessitating regulation of their intake. Nutritive sweeteners, such as sucrose, glucose, and fructose, provide ~4 kcal/g of energy, with excessive consumption linked to elevated risks of obesity and type II diabetes (Bray and Popkin, 2014). For instance, a standard 500 ml sweetened beverage contains about 55 g of sugar, surpassing the World Health Organization's recommended daily intake of 25 g when consumed in full (World Health Organization, 2015). Consequently, reducing the use of nutritive sweeteners in food products is essential to mitigate these health risks.

Reducing the amount of nutritive sweeteners diminishes the initial taste intensity and accelerates the decline in sweetness perception during consumption, compromising the enjoyment of taste throughout the food experience (DuBose et al., 1977; Rankin et al., 2009). Therefore, there is an increasing demand for strategies to enhance sweetness perception while minimizing nutritive sweetener intake.

Representative approaches to enhancing perceived sweetness without increasing nutritive sweetener intake include substituting nutritive sweeteners with high-intensity sweeteners (Chattopadhyay et al., 2014; Chadha et al., 2022) and employing chemical additives (Kumazawa and Kurihara, 1990; Vandenbeuch and Kinnamon, 2020; Frank and Byram, 1988; Sakai et al., 2001; Zhao et al., 2024). These methods amplify sweetness by chemically stimulating gustatory and olfactory receptors. However, repeated exposure to the same stimulus may lead to adaptation or habituation, reducing sweetness perception over time. Consequently, sweetness decreases during repeated consumption and swallowing within a food experience. To maintain sweetness and enhance the total perceived sweetness, it is essential to design dynamic taste stimuli that consider the dynamic nature of taste perception.

Accordingly, a method has been proposed to enhance sweetness perception without relying on high-intensity sweeteners or chemical additives by dynamically fluctuating the sugar concentration within food (Burseg et al., 2010, 2012). Existing methods activate sweetness-related neural activity by pulsing sugar concentration at ~5 s intervals. However, since these methods repeatedly present the same pattern of sugar concentration fluctuations, they fail to address habituation within central nervous system processes. To mitigate habituation, this study focuses on a novel approach involving the design of non-periodic sugar concentration changes throughout the entire eating process.

This study proposes a method to alleviate habituation by setting the initial sugar concentration below a baseline and gradually increasing it throughout the eating process. This approach enhances the total perceived sweetness while maintaining the same overall sugar content. By employing non-periodic sugar concentration changes, this method offers an alternative approach to mitigating habituation. In the future, it could be combined with existing methods, such as high-intensity sweeteners, chemical additives, or periodic sugar pulsation, to further enhance sweetness in food products.

**Figure 1:**
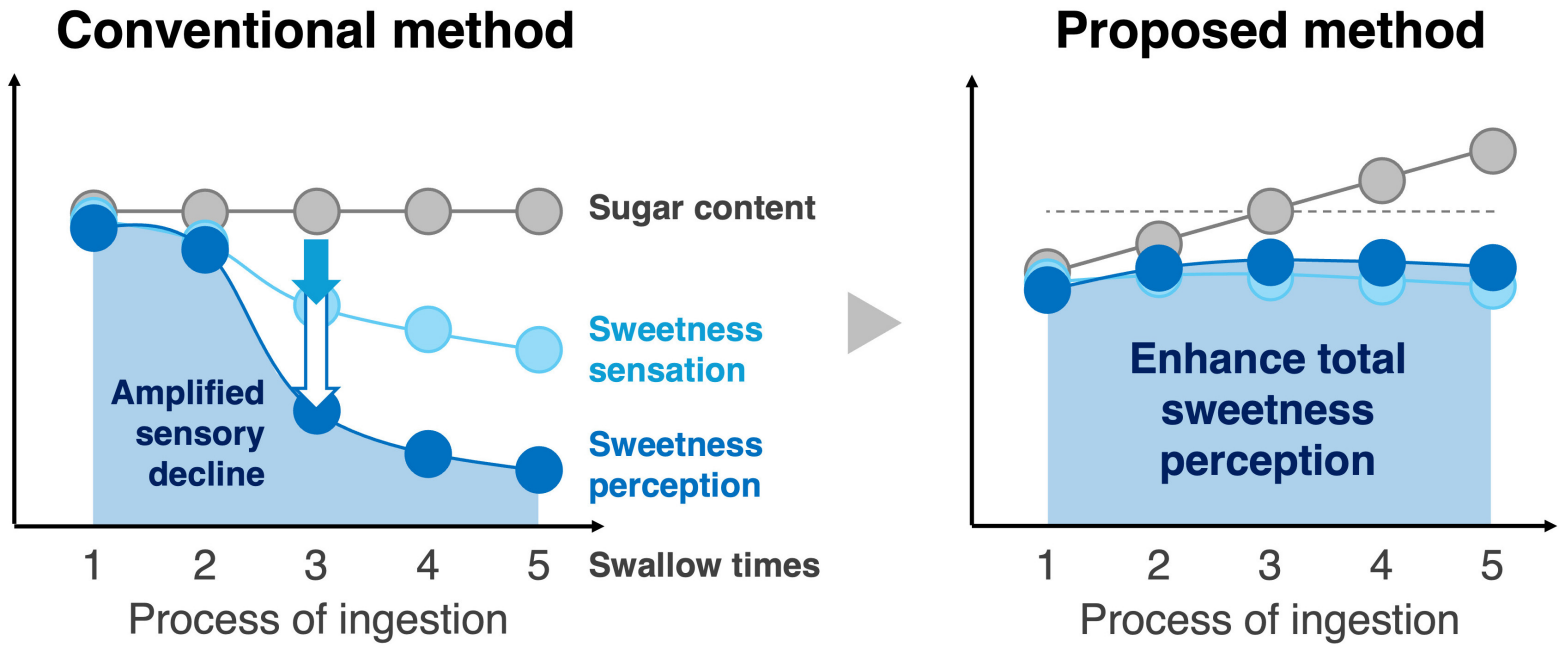
This study hypothesizes that a gradual increase in sugar concentration alleviates habituation and enhances total perceived sweetness without changing total sugar intake. Repeating a constant sugar concentration may amplify the perceived decline in sweetness due to rapid sensory adaptation. A gradual increase in sugar concentration could prevent this sharp decline, mitigate the reduction in sweetness perception, and improve total perceived sweetness.

This study hypothesizes that gradually increasing sugar concentration during the eating process alleviates habituation and enhances total perceived sweetness while maintaining sugar intake Figure 1. Repeated exposure to unchanging sweetness stimuli may amplify the decline in perception caused by adaptation, as perceptual differences in intensity become exaggerated (Lawless and Heymann, 2010). To counter this, the method starts with a sugar concentration below the baseline, gradually increases it, and ends above the baseline. This approach may prevent a rapid decline in sweetness perception and suppress its amplification, potentially enhancing total perceived sweetness without changing total sugar intake.

This study offers two key contributions:

1. Developed a device enabling gradual sugar concentration changes during the eating process and an evaluation application.
2. Demonstrated that, particularly in individuals prone to sweetness habituation, gradual increases in sugar concentration during the eating process can alleviate habituation and enhance total perceived sweetness without altering total sugar intake.

The aim of this study is to verify whether a gradual increase in sugar concentration during the eating process alleviates habituation and enhances total perceived sweetness without altering total sugar intake. To achieve this, the initial step involved determining the optimal starting sugar concentration. Subsequently, a device capable of presenting gradual sugar concentration changes and an application for sweetness evaluation were developed. Finally, sweetness evaluation experiments were conducted using these tools to assess the effects of gradual sugar concentration changes.

## 2 Related work

This chapter introduces methods for evaluating the taste of food, studies on the dynamic perceptual characteristics in response to repeated stimuli, and research on techniques for enhancing sweetness and saltiness.

### 2.1 Methods for evaluating the taste of food

Methods for evaluating the taste of food can be broadly categorized into post-hoc estimation, where evaluations are conducted after consumption (Dijksterhuis et al., 2014), and the Time-Intensity (TI) method, which involves real-time assessment during consumption (Busch et al., 2009; Wada et al., 2024).

Post-hoc estimation of taste intensity involves evaluating the taste of a target food after it has been consumed. This method allows for the assessment of the overall impression of the food, as it estimates taste intensity by weighting factors such as the initial value, peak intensity, and final value of the sequential taste profile. Since the evaluation is conducted only once after consumption, it is a relatively straightforward and easy-to-use approach. However, because this method does not capture the dynamic pattern of changes in taste intensity, it is not suitable for assessing the mitigation of habituation, which is the objective of this study.

The Time-Intensity (TI) method involves real-time evaluation of taste intensity while consuming the target food. This method is well-suited for assessing the mitigation of habituation, as it captures the dynamic changes in taste perception. Additionally, the time-integrated value of the taste intensity curve obtained through the TI method, known as the Area Under the Curve (AUC), serves as an effective indicator of the total perceived sweetness provided by the food.

In this study, the primary objective is to quantitatively evaluate the habituation phenomenon during the eating process. To achieve this, the Area Under the Curve (AUC) based on real-time evaluations using the TI method was adopted. This approach enables a detailed understanding of the dynamic progression of habituation across multiple eating and swallowing cycles, enabling the quantification of individual habituation characteristics. To minimize the impact of evaluation tasks on the measurements, the evaluation timing was standardized immediately after swallowing. Additionally, an intuitive evaluation method using a visual analog scale was employed.

### 2.2 Dynamic perceptual characteristics in response to repeated stimuli

When a constant sweetness stimulus is repeatedly presented, the perceived sweetness gradually declines over time (DuBose et al., 1977; Schiffman et al., 1994). This decrease in sweetness perception must be understood as the result of two distinct phenomena: adaptation at the receptor and sensory processing level, and habituation at the perceptual and cognitive level. This section discusses the characteristics and mechanisms of these phenomena, as well as the contextual effects that influence perceptual judgment.

#### 2.2.1 Mechanisms of taste adaptation and habituation

Taste adaptation refers to a decrease in sensitivity occurring along the pathway from peripheral sensory organs to the central nervous system. For sweetness, repeated binding and unbinding of sweet-tasting substances to sweet taste receptors (T1R2/T1R3) reduces the receptors' affinity for sweet molecules (Schiffman et al., 1994; Böhm et al., 1997). This phenomenon reflects a transient, functional change at the receptor level and is reversible, with sensitivity recovering once the stimulus is removed.

Habituation is characterized as a decrease in response at the perceptual and cognitive levels within the central nervous system. It is a neural mechanism in the sensory cortex that suppresses neuronal firing in response to repeated stimuli, enabling the efficient processing of novel stimuli (Kandel et al., 2021; Epstein et al., 2009).

#### 2.2.2 Contextual effects in continuous perception

The evaluation of physical quantities in the sensory cortex is influenced not only by the physical intensity of the stimulus but also by contextual biases (Lawless and Heymann, 2010). One notable bias is the contrast effect. The contrast effect refers to the phenomenon where a stimulus is perceived as stronger in the presence of a weaker stimulus and as weaker in the presence of a stronger stimulus. A key point is that the contrast effect can amplify perceptual differences beyond the actual physical differences, leading to an exaggerated sensory experience.

When adaptation occurs in response to repeated stimuli, a sharp decline in sweetness perception may trigger the contrast effect, amplifying the perceived reduction in sweetness and potentially accelerating habituation. To suppress this amplification of sweetness reduction caused by the contrast effect, this study proposes a method that gradually increases stimulus intensity in accordance with the eating process, thereby preventing abrupt declines in sweetness perception.

### 2.3 Methods for enhancing taste perception

Taste enhancement methods can be broadly classified into three categories: 1. Activation of gustatory and olfactory receptors using high-intensity sweeteners or chemical additives. 2. Activation of taste-related neural cells through the pulsation of sugar or salt concentrations at periodic intervals. 3. Manipulation of perceptual biases through the design of non-periodic changes in taste stimuli.

#### 2.3.1 Activation of gustatory and olfactory receptors using high-intensity sweeteners and chemical additives

Chemical substances that act on sweet taste receptors include high-intensity sweeteners and low concentrations of salt. High-intensity sweeteners bind to sweet taste receptors and elicit a strong sweet taste, serving as sugar substitutes (Chattopadhyay et al., 2014; Chadha et al., 2022). Additionally, low concentrations of salt activate sweet taste receptors, thereby enhancing sweetness (Kumazawa and Kurihara, 1990; Vandenbeuch and Kinnamon, 2020). Sweetness enhancement through olfactory stimulation has also been reported (Zhao et al., 2024). For example, the aroma of strawberries enhances the sweetness of whipped cream (Frank and Byram, 1988), while the scent of vanilla intensifies the sweetness of aspartame (Sakai et al., 2001). Since these methods only require the addition of chemical substances, they are easily applicable to actual food products. Moreover, because these substances are not metabolized, they have the potential to reduce the risk of obesity and type II diabetes.

However, the chemical substances added to enhance sweetness can also affect other taste and olfactory receptors, making it challenging to selectively enhance sweetness alone. In particular, certain high-intensity sweeteners are known to produce undesirable aftertastes or slight bitterness, depending on the type (Schiffman et al., 1995; Wiet and Beyts, 1992; Acevedo and Temussi, 2019). Additionally, if the concentration of salt is too high, it elicits a salty taste, necessitating precise concentration control.

Olfactory stimuli, such as strawberry or vanilla aromas, can alter the overall flavor profile, thereby limiting the range of applicable food products. Moreover, repeated exposure to the same gustatory or olfactory stimuli can induce adaptation or habituation. To sustain the taste and further increase the Area Under the Curve (AUC) of perceived sweetness, it is essential to introduce dynamic variations in the stimulus.

#### 2.3.2 Activation of taste neural cells through periodic pulsation of sugar and salt concentrations

The method of periodically pulsating sugar concentration within food to activate sweetness-related neural responses is known as pulse gustatory stimulation. The effectiveness of pulse gustatory stimulation was first demonstrated by Busch et al. through saltiness evaluation experiments using saline solutions (Busch et al., 2009). These findings have been applied to the development of reduced-salt bread (Noort et al., 2010) and the presentation of salty taste through electrical stimulation (Tsukamoto and Yamaguchi, 2017).

Pulsed gustatory stimulation has also been studied extensively in the context of sweetness. Burseg et al. demonstrated that pulsing the sugar concentration of a sucrose solution at ~5 s intervals during 40 s of consumption can enhance sweetness perception without altering the net sugar concentration (Burseg et al., 2010). Furthermore, it has been shown that sweetness enhancement occurs when the amplitude of sugar concentration fluctuations is set to at least Δ6% (ranging from 3 to 9%), using a 6% sucrose solution as the baseline (Burseg et al., 2012).

The mechanism of pulse gustatory stimulation is explained by the activation of taste-related neural responses (Burseg et al., 2010). When a taste stimulus is presented, taste neurons exhibit a transient response. This response occurs ~0.3 s to 2 s after stimulation with a high firing rate. Subsequently, the response shifts to a sustained response with a lower firing rate (Kandel et al., 2021). Pulse gustatory stimulation alternates between high-concentration and low-concentration stimuli in ~5 s cycles. This periodic fluctuation strengthens the transient high firing response, thereby enhancing the activation of taste-related neural pathways.

Pulse gustatory stimulation utilizes only the components inherently present in the food, thereby preventing alterations in taste quality and flavor. However, since the same stimulus pattern is repeated periodically, it fails to address habituation that arises in response to this repetitive pattern.

#### 2.3.3 Manipulation of perceptual bias through the design of non-periodic changes in taste stimuli

There are attempts to enhance taste by manipulating perceptual bias through non-periodic changes in the composition of food in accordance with the eating process.

Dijksterhuis et al. (2014) demonstrated that saltiness can be enhanced without changing the total amount of salt intake by varying the salt concentration in a high-zero-low pattern during the eating process. In their experiment, participants consumed a sandwich in three bites, with salt distribution patterns of 70%–0%–30% or 90%–0%–10%, which resulted in stronger perceived saltiness compared to the uniform distribution of 33%–33%–33%. This method leverages the concept of perceptual constancy based on human expectations. Since people generally expect the salt concentration to remain constant throughout the eating process, the reduced salt concentration in the second bite leads to an overestimation of saltiness. This approach is considered effective in situations where taste remains constant, as it does not rely on the occurrence of habituation.

In contrast, the gradual sugar concentration increase method proposed in this study aims to mitigate the sharp decline in sweetness perception caused by the contrast effect through alleviation of habituation. As such, this method is applicable in situations where habituation occurs. However, if the initial sugar concentration is set too low, perceptual constancy bias may arise, potentially leading to an underestimation of sweetness even as the sugar concentration increases. Therefore, to effectively enhance the AUC, it is crucial to avoid setting the initial sugar concentration excessively low.

## 3 Materials and methods

In this study, an experiment was designed to present a gradual increase in sugar concentration within food during the eating process, simulating a beverage with a variable concentration. This chapter describes the preparatory steps undertaken to verify the sweetness enhancement effect of the gradual sugar concentration increase. These steps include preliminary experiments for designing sugar concentration changes, selection of the food product, determination of flow rate, drinking duration, and swallowing intervals, development of a device for presenting sugar concentration fluctuation patterns, and creation of an application for sweetness evaluation.

### 3.1 Preliminary experiment for determining the initial sugar concentration

Since the sweetness perceived during a food experience is anchored to the sweetness of the first bite, the initial sugar concentration significantly influences the Area Under the Curve (AUC). Although the proposed method requires a lower initial sugar concentration, if the initial sweetness is too weak, the mitigation of habituation may not be sufficient to achieve an increase in AUC. Therefore, a preliminary experiment was conducted using commercially available sweetened beverages to determine an appropriate initial sugar concentration.

For the preliminary experiment, Calpis (manufactured by Asahi Soft Drinks) was used as the sweetened beverage. Calpis is a widely consumed sweetened beverage around the world and is one of the target drinks for sugar intake reduction. Since Calpis is typically consumed by diluting a concentrated syrup with water, its concentration can be easily adjusted. For these reasons, it was selected as a suitable beverage for the preliminary experiment. The baseline concentration of Calpis was set at 20%, following the manufacturer's recommended dilution ratio.

Five concentrations of Calpis were prepared at 12%, 16%, 20%, 24%, and 28%, corresponding to 0.6×, 0.8×, 1.0×, 1.2×, and 1.4 × of the baseline concentration, respectively. Participants rinsed their mouths with 15 ml of water before each trial, wore an eye mask, and consumed 15 ml of Calpis in a single sip. After each tasting, participants answered a questionnaire. This procedure was repeated for all five Calpis concentrations, presented in ascending order from the lowest to the highest concentration. The sweetness evaluation was conducted using a 9-point Likert scale, where 1 was labeled as “not sweet at all” and 9 as “extremely sweet.”

**Figure 2:**
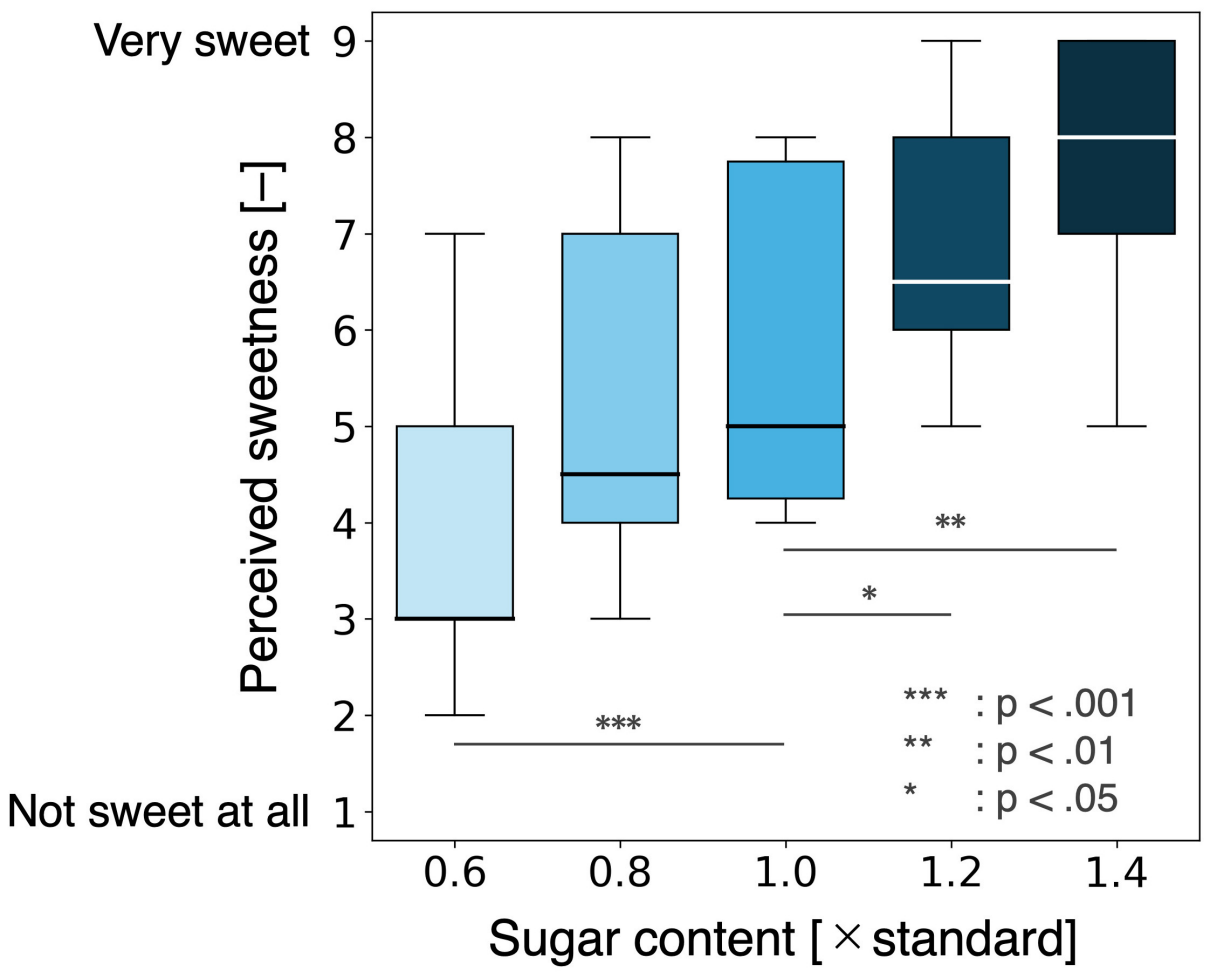
Sweetness evaluation for each concentration in a single sip. No significant difference in perceived sweetness was observed between the 0.8 × concentration and the baseline concentration. By setting the initial sugar concentration to 0.8 times the baseline, it is possible to prevent a decline in perceived sweetness throughout the food experience.

A total of 18 participants (13 males and five females) aged 21–44 years took part in the preliminary experiment. The sweetness evaluation scores obtained from the questionnaire are shown in Figure 2. The sweetness scores for each concentration were compared to the score for the 1.0 × concentration using the Wilcoxon signed-rank test, with p-values adjusted using the Benjamini-Hochberg method. The results of the tests are presented in Table 1. Significant differences (*p* < 0.05) were observed between 0.6 × −1.0×, 1.2 × −1.0×, and 1.4 × −1.0 × concentration pairs. In contrast, no significant difference was found between 0.8 × −1.0 × concentration pair (*p*≥0.05). In addition, effect sizes were calculated based on the statistics obtained from the Wilcoxon signed-rank test. Large effect sizes (|*r*|≥0.5) were observed for the comparisons between 0.6 × −1.0×, 1.2 × −1.0×, and 1.4 × −1.0 × . However, the effect size for the comparison between 0.8 × −1.0 × was moderate (0.3 ≤ |*r*| <0.5).

**Table 1:** The results of the Wilcoxon signed-rank test for sweetness differences between concentrations.

**Pair**	**BH-adjusted *p*-value**	**Effect size (*r*)**
0.6 × −1.0 ×	7.62 × 10^−4^^***^	0.795
0.8 × −1.0 ×	1.37 × 10^−1^	0.407
1.2 × −1.0 ×	1.58 × 10^−2^^*^	0.713
1.4 × −1.0 ×	1.20 × 10^−3^^**^	0.879

*p < 0.05, **p < 0.01, and ***p < 0.001.

From these results, it can be concluded that a sugar content at 0.8 times the baseline level is less likely to be perceived as different in sweetness from the baseline. Therefore, reducing the initial sugar content to 0.8 times the baseline may help prevent an overall decline in sweetness perception throughout the food experience. However, since the preliminary experiment included a higher proportion of male participants, the optimal initial concentration might differ in more gender-balanced samples.

### 3.2 Selection of food products

In the preliminary experiment, the goal was to design an appropriate initial concentration to prevent AUC reduction, making the use of Calpis suitable. However, for the main experiment, it is necessary to evaluate the phenomenon of sweetness habituation. Since Calpis also includes perceptible elements such as sourness and viscosity, it may not be appropriate for this purpose.

In contrast, sucrose solution is widely used as a standard substance in prior research evaluating sweetness and presents a pure sweet taste, eliminating the influence of other taste qualities (Mao et al., 2019). Therefore, sucrose solution was selected as the food product for verifying the sweetness enhancement effect of the proposed method.

The sugar concentration of the sucrose solution was initially designed with 5% as the baseline concentration, considered approximately equivalent in sweetness to 20% diluted Calpis. Based on this baseline, the initial and final sugar concentrations were planned to be 4% (0.8 times the baseline) and 6% (1.2 times the baseline), respectively, with a linear change throughout the eating process.

However, as will be described in Section 3.4.2, verification of the DynaDrink system revealed that the actual output concentration stabilized at ~94% of the intended target under constant conditions. Therefore, the effective baseline sugar concentration was 4.7%, and the actual sugar concentration transitions were from 3.8 to 5.6% in the increasing condition and from 5.6 to 3.8% in the decreasing condition.

### 3.3 Flow rate, drinking duration, and swallowing interval

In this study, the flow rate was set to 130 ml/min, the drinking duration to 15 s, and the swallowing interval to 3 s. These parameters were determined based on the ease of sweetness evaluation and the total sugar intake throughout the experiment.

To ensure sufficient sweetness perception and facilitate evaluation, it was necessary to provide an adequate flow rate of the sweetened beverage. A flow rate of 130 ml/min was chosen to allow the sweetened beverage to sufficiently spread within the oral cavity during each swallow while maintaining ease of swallowing. The swallowing interval was set to 3 s to ensure that participants had enough time to perceive the sweetness of each sip.

The experiment involved six drinking sessions, including practice trials. To limit the total sugar intake, the drinking duration for each session was set to 15 s, resulting in a total sugar intake of 10.2 g for the entire experiment. This intake is well below the World Health Organization's recommended daily sugar intake of 25 g (World Health Organization, 2015).

### 3.4 Sugar concentration fluctuation presentation device (DynaDrink)

**Figure 3:**
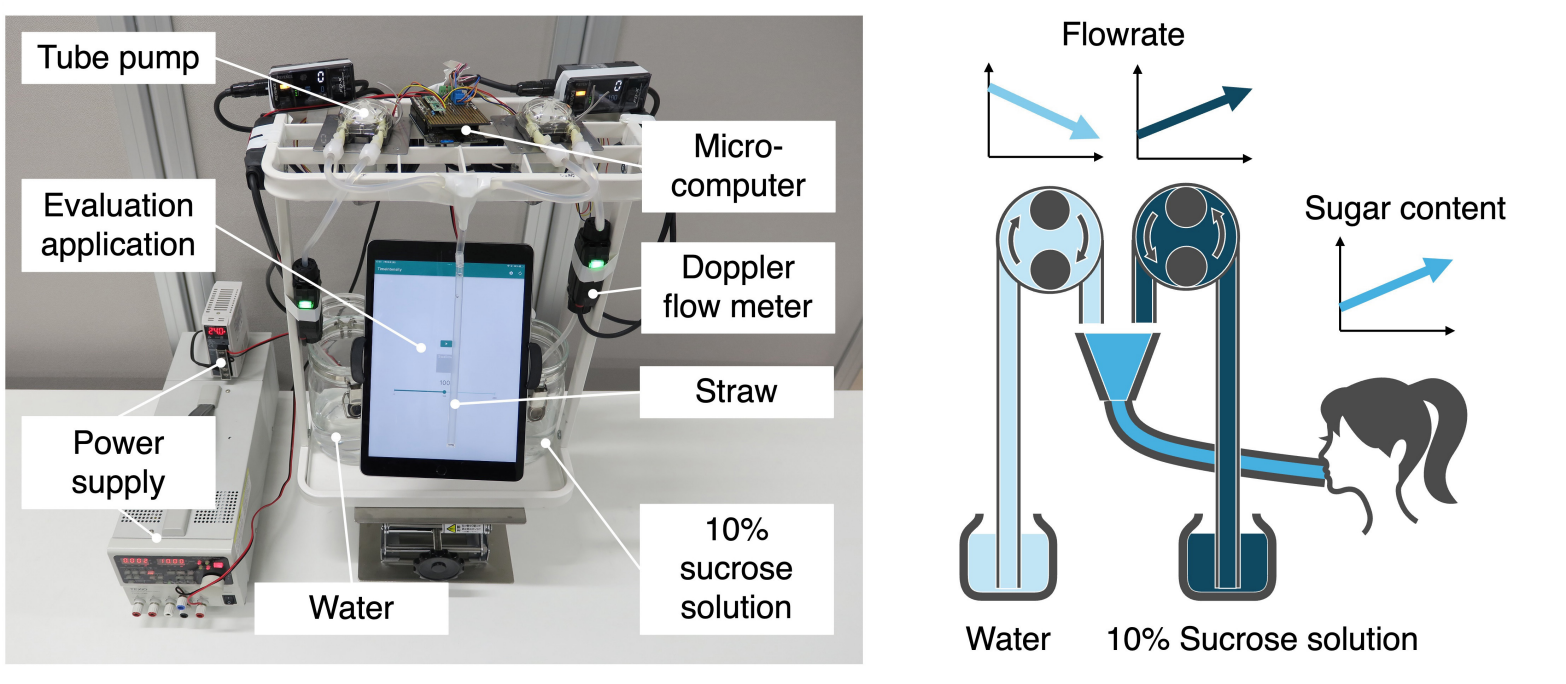
The DynaDrink system outputs sucrose solutions with varying sugar concentrations by combining water and sucrose solution at different flow rates. By adjusting the flow rates of the two liquids, the system produces sucrose solutions with dynamically changing sugar concentrations, allowing for the presentation of gradual fluctuations in accordance with the eating process.

To present the gradual increase in sugar concentration of sucrose solution according to the eating process, a sugar concentration fluctuation presentation device, named DynaDrink, was developed Figure 3.

#### 3.4.1 Design and implementation of DynaDrink

The development requirements for the sugar concentration fluctuation presentation device, DynaDrink, were to enable continuous variation of sugar concentration while maintaining constant values for all other parameters. To achieve continuous sugar concentration variation, a 10% sucrose solution and water were pumped independently using two separate pumps. By controlling the rotational speed of each pump, the mixing ratio of the two liquids was continuously adjusted, allowing for fluctuations in the sugar concentration around 5%. To ensure that parameters other than sugar concentration remained unchanged, the total flow rate of the 10% sucrose solution and water was kept constant. This control mechanism maintained a steady flow rate of the mixed solution, ensuring that only the sugar concentration was varied.

The 10% sucrose solution was prepared by mixing natural mineral water (I Lohas by Coca-Cola) with granulated sugar (Suzuran Co., Ltd.). To accurately control the flow rates of the 10% sucrose solution and water, a tube pump driven by a stepping motor (WPX1-NF4.8FA2-W6-CRP, manufactured by Wellco) was used. The rotation speed of the pump was precisely controlled by a microcomputer (Sony Spresense) to achieve accurate and stable flow rate control.

#### 3.4.2 Performance evaluation experiment for DynaDrink

A performance evaluation experiment was conducted for the sugar concentration fluctuation presentation device (DynaDrink) using the settings to be employed in the main experiment.

The sucrose solution profiles are shown in Table 2. Three sugar concentration transition conditions were established: constant (Baseline), increasing (Proposed Method), and decreasing (Inverse of the Proposed Method). For all conditions, the target average sugar concentration was set at 5%, and the target total flow rate was set at 130 ml/min.

**Table 2:** Stimulus design profile for each condition.

**Condition**	**Constant**	**Increase**	**Decrease**
Beverage	Average 5% sucrose solution		
Flow rate	130 ml/min		
Duration of drinking	15 s		
Swallowing interval	3 s		
10% sucrose solution flowrate	65 ml/min	52–78 ml/min	78–52 ml/min
Water flowrate	65 ml/min	78–52 ml/min	52–78 ml/min
Sugar content	Stay 5%	4%–6%	6%–4%

In the constant condition, both the 10% sucrose solution and water were continuously output at 65 ml/min each, resulting in a mixed solution with a fixed sugar concentration of 5% (baseline sugar concentration).

In the increasing condition, the flow rate of the 10% sucrose solution was increased from 52 to 78 ml/min, while the flow rate of water was decreased from 78 to 52 ml/min. This adjustment produced a mixed solution with a sugar concentration that increased linearly from 4% (0.8 times the baseline) to 6% (1.2 times the baseline). These sugar concentration changes were determined based on the results of the preliminary experiment (Section 3.1).

In the decreasing condition, the operations were reversed, with the sugar concentration gradually declining from 6 to 4%.

To evaluate the performance of DynaDrink, it was operated according to the target conditions for 25 trials each under the constant, increasing, and decreasing conditions. The flow rates of the 10% sucrose solution and water were measured using an ultrasonic Doppler flow meter (FD-XS8, Keyence), and the sugar concentration was estimated based on the measured flow rates.

**Figure 4:**
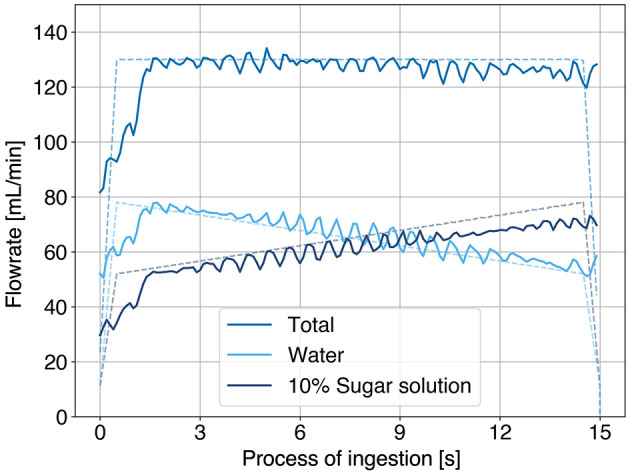
Flow rate of 10% sucrose solution, water, and total flow rate under the increasing condition.

**Figure 5:**
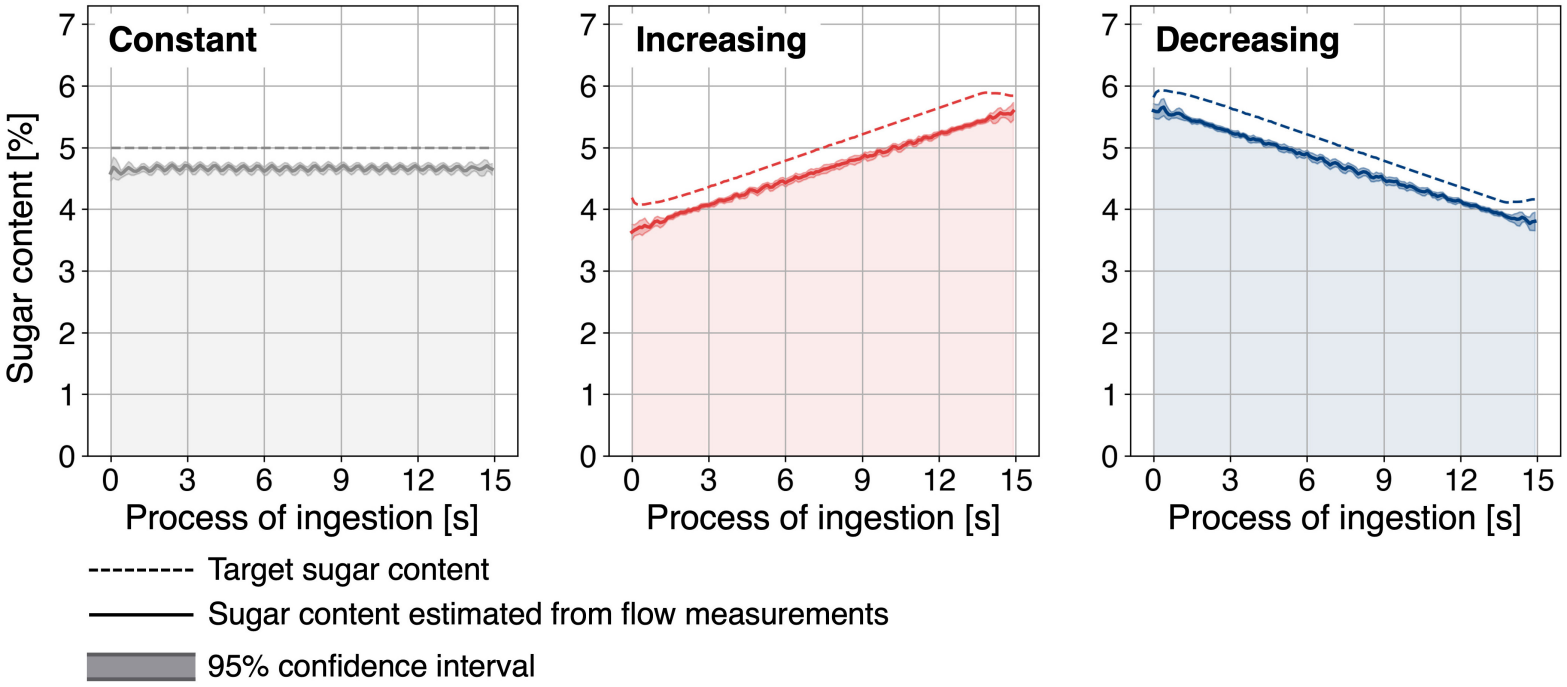
Estimated sugar concentration transition for each condition based on measured flow rates.

The flow rate measured during a single trial under the increasing condition is shown in Figure 4. The sugar concentration estimated from the flow meter measurements for each condition is presented in Figure 5. Across all conditions, the average sugar concentration was 4.7% (93% of the target value), and the total flow rate was 122 ml/min (94% of the target value). This discrepancy was attributed to the average flow rate of the 10% sucrose solution pump being 56 ml/min (88% of the target setting).

Nevertheless, by setting the baseline sugar concentration to 4.7%, it was confirmed that the sugar concentration could be varied from 0.8 times to 1.2 times the baseline, as intended.

### 3.5 Sweetness evaluation application

**Figure 6:**
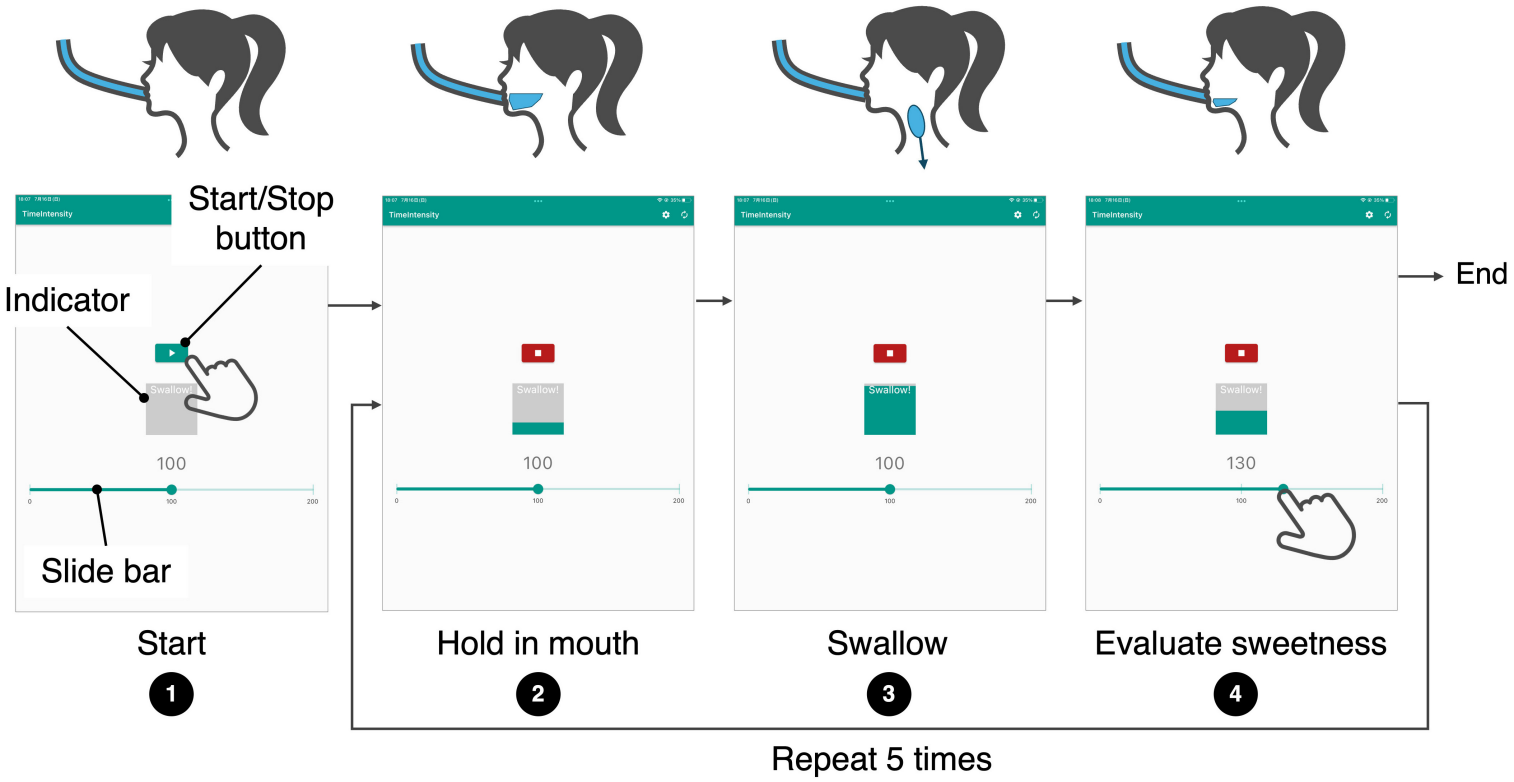
Operation flow of the sweetness evaluation application. Disp. 1: When the start button is pressed, the DynaDrink device begins operation. Disp. 2: During the 3-s period in which the central indicator gradually fills, participants hold the beverage in their mouths. Disp. 3: Once the indicator is fully filled, participants swallow the beverage. Disp. 4: Immediately after swallowing, participants tap on the slider bar at the bottom of the screen to evaluate the sweetness. The sequence from Disp. 2 to Disp. 4 is repeated five times, after which the DynaDrink device stops.

To evaluate sweetness in accordance with the eating process, a dedicated iPad application was developed Figure 6. The key development requirements were to regulate the timing of swallowing and evaluation, and to enable immediate sweetness assessment. To regulate the timing of swallowing and evaluation, an indicator was displayed at the center of the screen. The indicator filled up every 3 s, corresponding to the swallowing interval. Participants were instructed to swallow when the indicator became full and to immediately perform the sweetness evaluation thereafter. For immediate sweetness evaluation, a Visual Analog Scale (VAS) was displayed on the screen. Participants could record their sweetness rating with a single tap on the scale. The slider bar was labeled “0” (no sweetness perceived), “100” (baseline sweetness), and “200” (twice the baseline sweetness), with these labels explained beforehand.

## 4 Experimental design

The hypothesis of this study is that a gradual increase in sugar concentration in accordance with the eating process mitigates habituation and enhances the Area Under the Curve (AUC) of perceived sweetness. To verify this hypothesis, an experiment was conducted using the food product, sugar concentration fluctuation presentation device, and sweetness evaluation application described in 3. Participants consumed the beverage under the specified drinking volume, flow rate, and sugar concentration transition conditions, and evaluated the sweetness using the developed application. The experiment was conducted using a within-subjects design.

### 4.1 Experimental conditions

Three sugar concentration transition conditions were tested Figure 5.

- Constant condition (baseline): the sucrose solution was presented at a fixed concentration of 4.7% (baseline concentration) throughout the entire process.
- Increasing condition (proposed method): the concentration increased linearly from 3.8% (0.8 times the baseline) at the start to 5.6% (1.2 times the baseline) at the end.
- Decreasing condition (inverse of the proposed method): the concentration decreased linearly from 5.6% (1.2 times the baseline) at the start to 3.8% (0.8 times the baseline) at the end.

In all conditions, the sucrose solution had an average sugar concentration of 4.7% and a flow rate of 122 ml/min. The sugar concentration changes for the increasing and decreasing conditions were determined based on the results of the preliminary experiment (Section 3.1).

The effectiveness of the proposed method was verified by comparing the constant condition with the increasing condition. To further investigate the impact of the direction of sugar concentration change on sweetness enhancement, the decreasing condition was included as a symmetrical counterpart to the increasing condition. In particular, under the decreasing condition, the initial sugar content is high, which may enhance sweetness similarly to the high-zero-low salt concentration variation method due to perceptual constancy (Dijksterhuis et al., 2014). This effect was examined by comparing the decreasing condition with the constant condition.

### 4.2 Experimental process

The experiment consisted of three continuous drinking conditions with five swallows each, followed by a post-experiment questionnaire.

**Figure 7:**
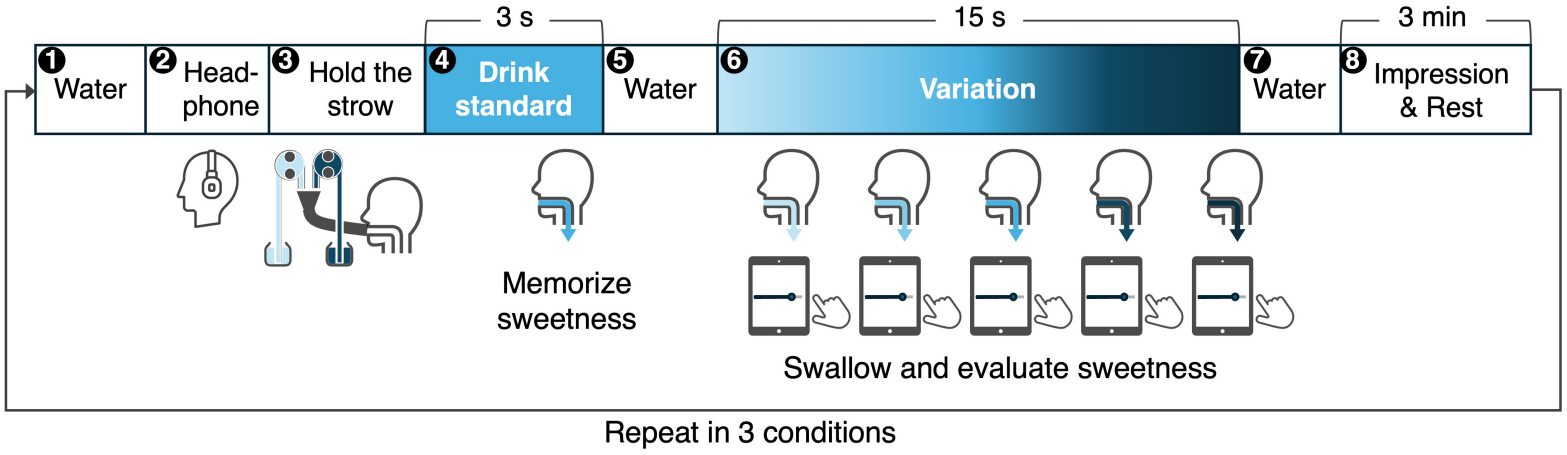
Procedure for continuous drinking and sweetness evaluation.

**Figure 8:**
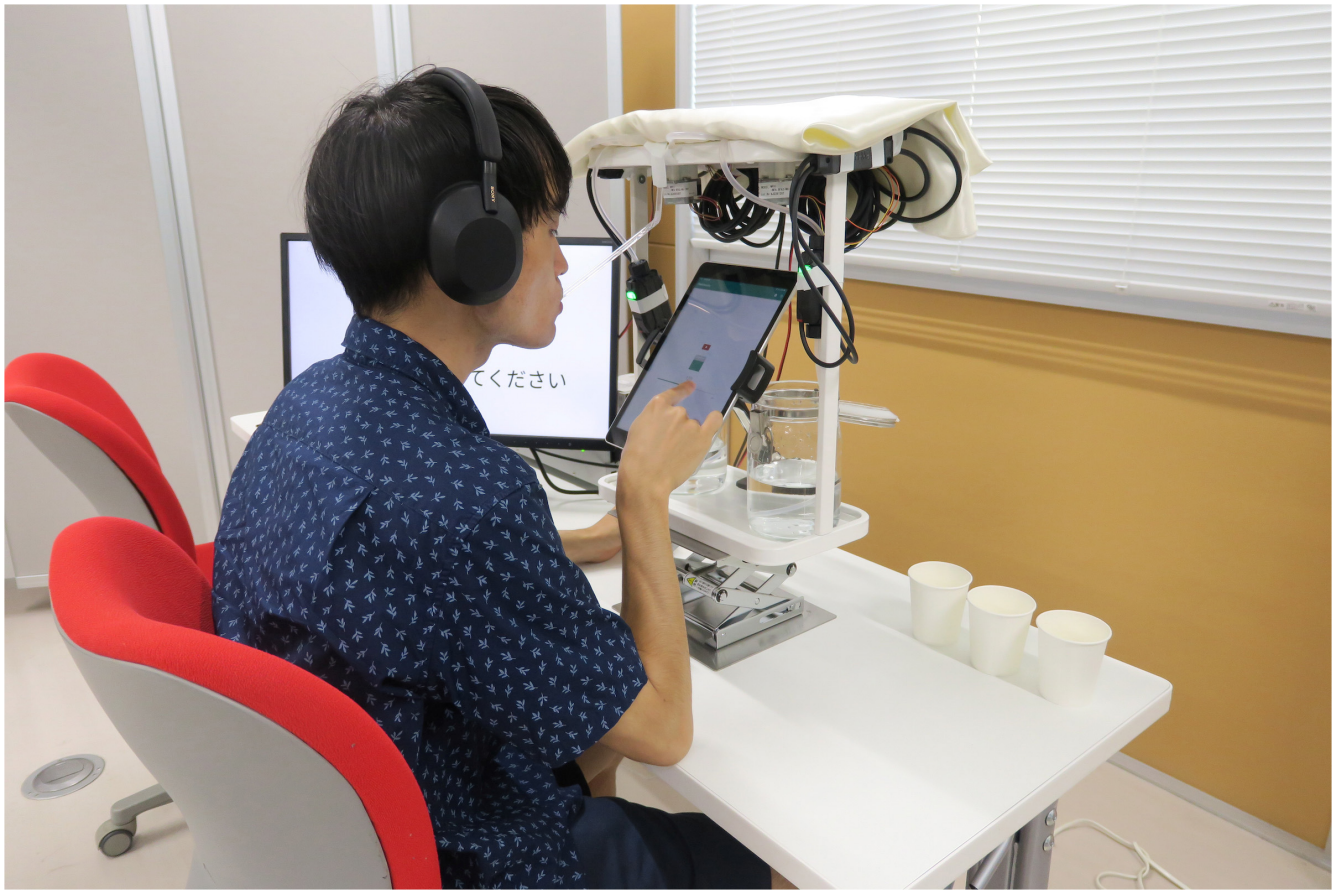
Sweetness evaluation during drinking using DynaDrink.

The procedure for continuous drinking was as follows Figure 7:

Step 1: Participants took a small amount of water into their mouths, rinsed, and swallowed it.
Step 2: Participants wore noise-canceling headphones (Sony WH-1000XM5) set to noise-canceling mode. White noise was played through the headphones to block sounds from the DynaDrink device.
Step 3: Participants inserted a straw attached to the DynaDrink outlet into their mouths at a 15-degree downward angle from horizontal. They bit the straw about 1 cm from the tip and adjusted their head position so that the straw was parallel to their tongue. This position ensured that the sucrose solution flowed evenly over the entire tongue and was not obstructed by the straw.
Step 4: A baseline sucrose solution was provided from DynaDrink for 3 s. Participants held the sucrose solution in their mouths for 3 s and then swallowed it. This sip was used as a reference for the baseline sweetness, which participants were asked to remember for later evaluations.
Step 5: Participants rinsed their mouths with a small amount of water, then reinserted the DynaDrink straw into their mouths.
Step 6: DynaDrink dispensed sucrose solution for 15 s. Participants swallowed the solution at 3 s, 6 s, 9 s, 12 s, and 15 s, following instructions displayed on an iPad attached to DynaDrink (Figure 8). After each swallow, participants evaluated the sweetness of the sucrose solution using a Visual Analog Scale (VAS) displayed on the iPad. The VAS was labeled with values from 0 (no sweetness) to 200 (twice as sweet as the baseline), with 100 representing the baseline sweetness.
Step 7: Participants rinsed their mouths with a small amount of water to remove any residual sucrose solution.
Step 8: Participants orally described their impressions of the sweetness transition during **Step 6** and then took a 3 min break. During the break, participants viewed nature scenery videos^1^ on a monitor, which was intended to promote dishabituation and return participants to a psychologically neutral state.

This procedure was repeated for each of the three experimental conditions described in Section 4.1. The order of the three conditions was randomized for each participant to counterbalance any order effects.

After completing all the conditions, participants were asked to complete a questionnaire. The survey collected personal information (age, gender) as well as information on participants' frequency of sweetened beverage consumption and their preferences for sweetness in such beverages.

### 4.3 Data analysis

In this experiment, participants were classified based on their susceptibility to habituation under the constant condition. The hypothesis of this study is that mitigation of habituation leads to an increase in the Area Under the Curve (AUC). Therefore, it is expected that participants who are more susceptible to habituation under the constant condition will exhibit a stronger AUC enhancement effect under the proposed method, whereas participants who are less susceptible to habituation will not show a significant effect. To verify this hypothesis, participants were grouped according to their susceptibility to habituation under the constant condition. The AUC was then compared between the constant condition and the increasing condition for each group.

The presence or absence of habituation under the constant condition is reflected in the dynamic characteristics of the sweetness transition data. To capture these dynamic features, this study employs B-spline interpolation to functionalize the discrete time-series data of sweetness transitions, followed by functional principal component analysis (fPCA). In sensory evaluation using the Time-Intensity (TI) method for food analysis, B-spline interpolation is commonly used to functionalize time-series data to extract dynamic information (Ledauphin et al., 2005). By applying fPCA to the functionalized sweetness transition data, it is possible to extract principal component functions that capture variations at different time scales, thereby enabling the evaluation of habituation.

The procedure for participant classification using functional principal component analysis (fPCA) is as follows: First, sweetness transition data under specific conditions was transformed into functional form using B-spline interpolation. The B-spline basis functions are determined by control points, order, and knot vectors. For the time-series data consisting of five points at intervals between 3 s and 15 s, the number of control points was set to 5. To prevent overfitting, the order of the basis functions was set to 3. The knot vector, fixed at the endpoints and uniformly weighted for each control point, was defined as [3, 7, 11, 15]. Next, functional principal component analysis was applied to the functionalized sweetness transition data to extract principal component functions. Each principal component function captures specific dynamic features on varying time scales. In this study, to examine the habituation characteristics throughout the eating process, a principal component function demonstrating a monotonic decrease during the eating process was selected as indicative of habituation. Finally, participants were classified into habituated and non-habituated groups based on the sign (positive or negative) of the coefficients of the principal component function reflecting habituation characteristics.

After grouping, the AUC was calculated and compared between the increasing and constant conditions as well as between the decreasing and constant conditions within each group. The AUC was computed by multiplying the sweetness evaluation values obtained in Step 6 (Section 4.2) by the interval time of 3 s and summing the results across five evaluations.

Following confirmation of data normality and homoscedasticity, paired *t*-tests were conducted. To address the issue of multiplicity, *p*-values were adjusted using the Benjamini–Hochberg method. The significance level was set at 5%. Additionally, effect sizes were calculated as *r* values.

In this data analysis, individual differences in habituation tendencies were addressed by classifying participants based on their habituation profiles. Furthermore, by performing paired *t*-tests within each group, it was possible to obtain statistically clear results regarding the effects of sugar concentration patterns, even in the presence of individual variability in AUC.

### 4.4 Statistical hypotheses

The hypotheses tested in this experiment are as follows:

H1 Inc. > Const. (habituated): in the habituated group, AUC under the increasing condition is higher than under the constant condition.
H2 Inc. ≈ Const. (non-habituated): in the non-habituated group, there is no significant difference in AUC between the increasing and constant conditions.
H3 Dec. <Const. (habituated): in the habituated group, AUC under the decreasing condition is lower than under the constant condition.
H4 Dec. > Const. (non-habituated): in the non-habituated group, AUC under the decreasing condition is higher than under the constant condition.

In the habituated group, it was hypothesized (H1) that the gradual increase in sugar content would mitigate the sharp decline in sweetness observed under constant conditions, thereby alleviating habituation and increasing the AUC. Conversely, in the non-habituated group, since no decline in sweetness occurs under constant conditions, it was predicted (H2) that the gradual increase in sugar content would have a limited sweetness-enhancing effect.

Additionally, in the habituated group, it was hypothesized (H3) that the gradual decrease in sugar content would lead to a more pronounced decline in sweetness, intensifying the contrast effect and reducing the AUC. In the non-habituated group, the high initial sugar content in the decrease condition was expected to produce an effect similar to the high-zero-low method due to perceptual constancy, potentially increasing the AUC (H4) (Dijksterhuis et al., 2014).

### 4.5 Participants

This experiment involved 25 participants (14 males, 11 females) aged 22–50 years. Participants refrained from consuming any food or beverages other than water for three hours prior to the experiment. Additionally, they avoided drinking water for one hour before the session.

Before the main experiment, participants practiced drinking and evaluating using the DynaDrink device and the sweetness evaluation application three times to ensure familiarity with the process. The experiment was conducted only after participants were adequately accustomed to the procedure.

This study was approved by the Research Ethics Committee of the University of Tokyo (Approval No. 24-068) and conducted with informed consent obtained from all participants.

## 5 Results

### 5.1 Grouping of participants based on habituation tendency

To investigate the presence or absence of habituation in each individual under the constant condition, participants were grouped based on the dynamic characteristics of their perceived sweetness data in the constant condition.

**Figure 9:**
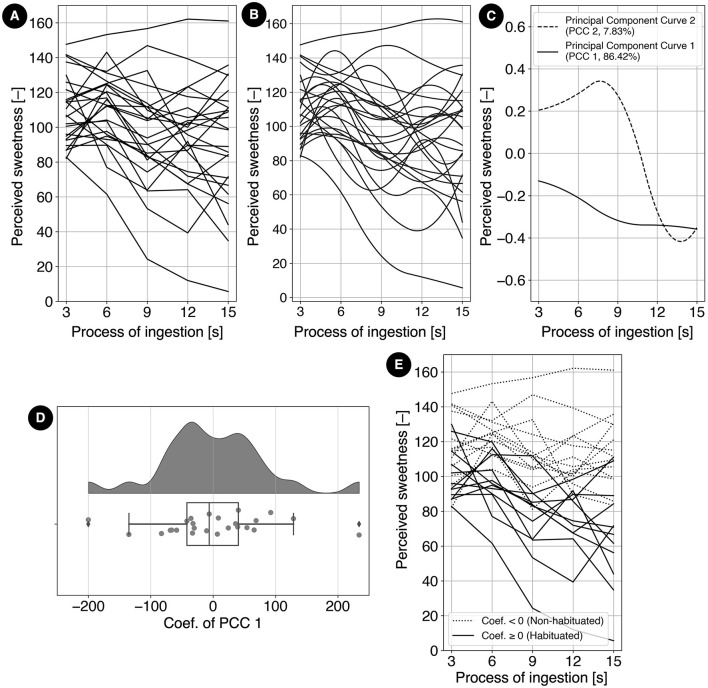
**(A)** Sweetness transitions for each participant under the constant condition. **(B)** Functionalized sweetness transitions for each participant, derived from **(A)** using B-spline interpolation to extract dynamic characteristics. **(C)** The first and second principal component functions extracted from the functional group in **(B)** through functional principal component analysis. The proportions of explained variance are 86.4% and 7.83%, respectively. The first principal component function, characterized by a monotonic decrease, represents habituation characteristics throughout the eating process. **(D)** Distribution of coefficients for the first principal component function representing habituation characteristics. The distribution forms two peaks divided by zero, separating participants into a habituated group (positive coefficients) and a non-habituated group (negative coefficients). **(E)** Sweetness transitions under the constant condition for each participant, classified into two groups based on the sign of the first principal component function coefficients. The habituated group consists of 12 participants, while the non-habituated group consists of 13 participants.

The transitions of perceived sweetness for all 25 participants under the constant condition are shown in Figure 9A. These data were transformed into functional form using B-spline interpolation, as illustrated in Figure 9B. From the functionalized transitions of perceived sweetness, principal component functions were derived through functional principal component analysis (fPCA). The first and second principal component functions, which collectively explained over 90% of the variance, are presented in Figure 9C. The first principal component function, with an explanatory proportion of 86.4%, exhibited a sharp decline from 3 s to 9 s, followed by a more gradual decrease beyond 9 s. The second principal component function, accounting for 7.83% of the variance, demonstrated an increase from 3 s to 9 s, followed by a rapid decline. Based on these characteristics, the first principal component function was identified as representing habituation characteristics throughout the eating process.

The distribution of the coefficients for the first principal component function, which reflects habituation characteristics throughout the eating process, is shown in Figure 9D. A positive coefficient indicates a tendency toward habituation under the constant condition, whereas a negative coefficient suggests a propensity for sensitization (the opposite of habituation). The distribution is bimodal, divided into two peaks around zero, suggesting that the participants can be classified into two distinct groups. Based on this observation, 12 participants with positive coefficients were categorized into the habituated group, while 13 participants with negative coefficients were categorized into the non-habituated group.

The transitions of perceived sweetness under the constant condition for the 25 participants are shown in Figure 9E. Participants were divided into two groups based on the coefficients of the first principal component function. The habituated group (12 participants) had positive coefficients, while the non-habituated group (13 participants) had negative coefficients.

The habituated group exhibited lower overall sweetness perception under the constant condition, with a declining trend from 3 to 15 s, suggesting greater susceptibility to habituation. In contrast, the non-habituated group showed higher overall sweetness perception and greater resistance to habituation.

Although individual differences in sweetness intensity were observed, the classification by temporal response patterns allowed for subsequent group-wise comparison.

### 5.2 Comparison between increasing and constant conditions

**Figure 10:**
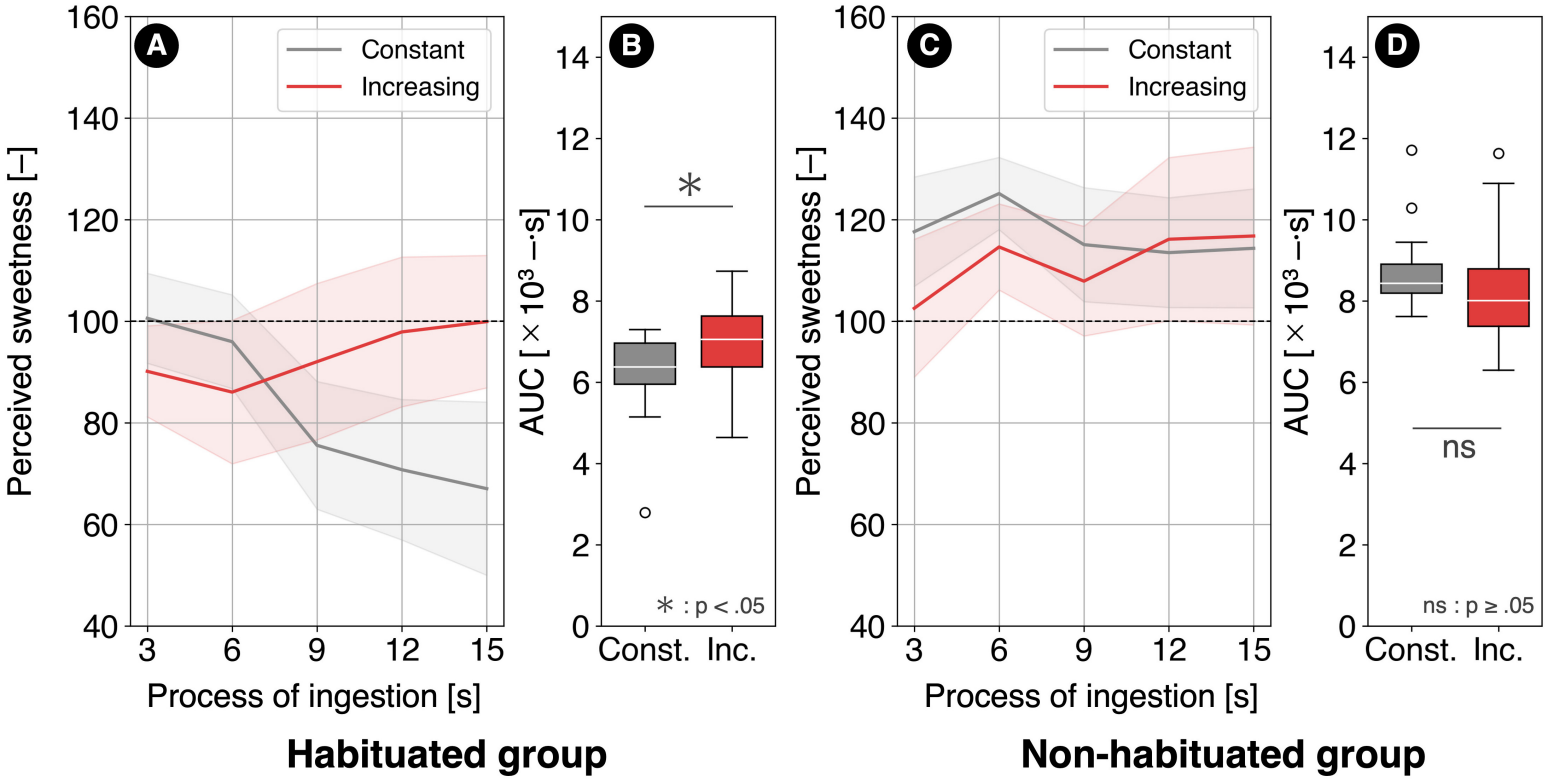
**(A)** Sweetness transitions under the increasing and constant conditions for the habituated group and **(B)** their AUCs, as well as **(C)** sweetness transitions under the increasing and constant conditions for the non-habituated group and **(D)** their AUCs.

The sweetness transitions under the increasing and constant conditions for the habituated group (12 participants) are shown in Figure 10A. In this group, the AUC under the increasing condition was significantly higher than that under the constant condition [(*t*_(11)_ = 3.27, *p* = 0.0294, *r* = 0.702)], as illustrated in Figure 10B. The average increase in AUC was 13.6% relative to the constant condition, with 83.3% of participants exhibiting an increase in AUC under the increasing condition.

The sweetness transitions under the increasing and constant conditions for the non-habituated group (13 participants) are shown in Figure 10C. In this group, no significant difference in AUC was observed between the increasing and constant conditions [(*t*_(12)_ = –1.53, *p* = 0.301, *r* = 0.405)], as illustrated in Figure 10D. The average change in AUC was −4.73% relative to the constant condition, with only 23.0% of participants showing an increase in AUC under the increasing condition.

### 5.3 Comparison between decreasing and constant conditions

**Figure 11:**
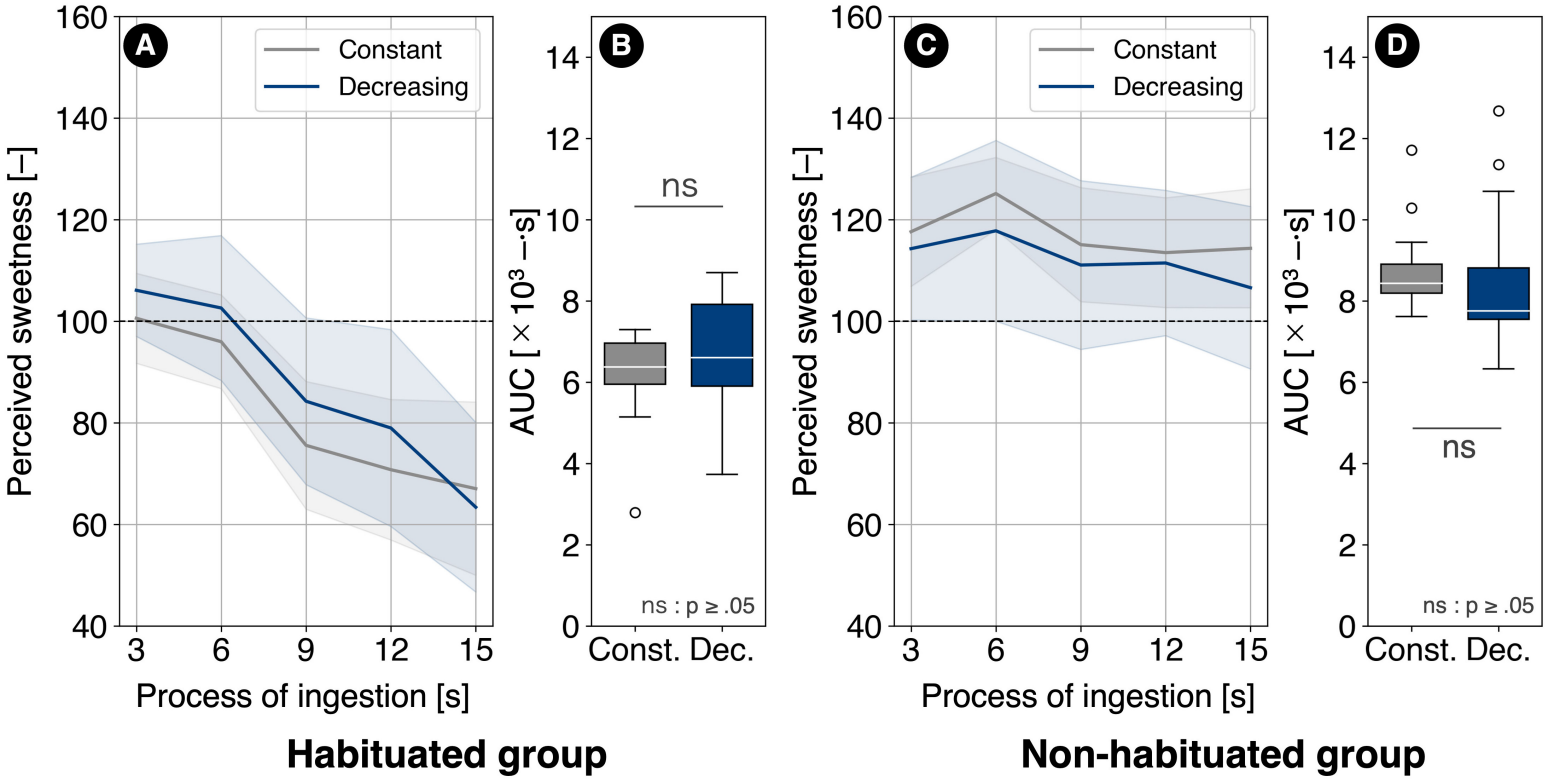
**(A)** Sweetness transitions under the decreasing and constant conditions for the habituated group and **(B)** their AUCs, as well as **(C)** sweetness transitions under the decreasing and constant conditions for the non-habituated group and **(D)** their AUCs.

The sweetness transitions under the decreasing and constant conditions for the habituated group (12 participants) are shown in Figure 11A. In this group, no significant difference in AUC was observed between the decreasing and constant conditions [(*t*_(11)_ = 0.992, *p* = 0.381, *r* = 0.286)], as illustrated in Figure 11B. The average increase in AUC was 6.19% relative to the constant condition, with 66.6% of participants exhibiting an increase in AUC under the decreasing condition.

The sweetness transitions under the decreasing and constant conditions for the non-habituated group (13 participants) are shown in Figure 11C. In this group, no significant difference in AUC was observed between the decreasing and constant conditions [(_*t*(12)_ = −0.907, *p* = 0.381, *r* = 0.253)], as illustrated in Figure 11D. The average change in AUC was −4.18% relative to the constant condition, with 30.7% of participants exhibiting an increase in AUC under the decreasing condition.

### 5.4 Results of the subjective questionnaires and post-experiment questionnaires

**Figure 12:**
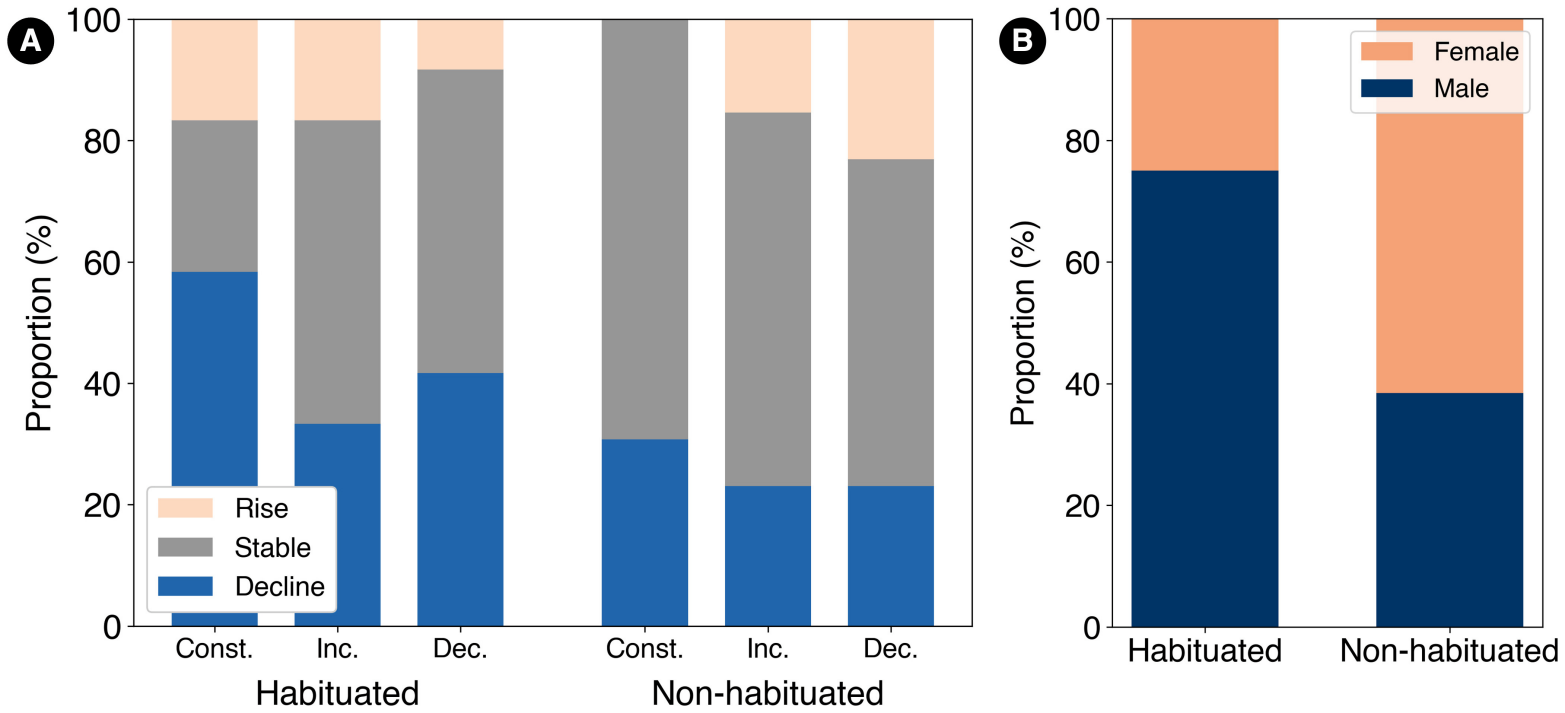
**(A)** Impressions of sweetness transitions reported after consumption. The proportion of participants reporting a decrease in sweetness was reduced to approximately half in the habituated group between the constant and increasing conditions, while the change was minimal in the non-habituated group. **(B)** Gender ratio by group. The habituated group had a male ratio of 75.0%, whereas the non-habituated group had a male ratio of 38.4%.

The impressions of sweetness transitions reported after consumption (step 8) are shown in Figure 12A. Regarding the proportion of participants who reported a decrease in sweetness, the habituated group showed a reduction in this proportion between the constant and increasing conditions, while the non-habituated group exhibited almost no change.

The gender ratio for each group, derived from the post-experiment questionnaire, is shown in Figure 12B. The habituated group had a male ratio of 75.0%, whereas the non-habituated group had a male ratio of 38.4%. A chi-squared test was conducted to examine this difference in gender ratios, revealing no statistically significant difference [χ^2^(1, *N* = 25) = 2.06, *p* = 0.15]. However, the effect size, as measured by Cramér's *V* =.29, suggests a small to approaching-medium association according to Cohen's criteria (Cohen, 2013).

## 6 Discussion

Under constant conditions, the temporal changes in perceived sweetness varied greatly across participants Figure 9A. However, functional principal component analysis (fPCA) enabled the extraction of principal component functions reflecting habituation characteristics, allowing participants to be classified into two groups based on the sign of their coefficients.

Regarding the AUC under the increasing condition, the habituated group (12 participants) exhibited a significantly higher AUC compared to the constant condition, while no significant difference was observed in the non-habituated group (13 participants). This supports hypotheses H1 [Inc. > Const. (habituated)] and H2 [Inc. ≈ Const. (non-habituated)]. Classification based on habituation characteristics and the use of paired *t*-tests enabled the detection of statistically significant effects beyond individual differences in AUC.

In the habituated group, a sharp decrease in sweetness was observed between 6 and 9 s under the constant condition. This suggests that the amplification of sweetness reduction due to the contrast effect occurred as hypothesized. Under the increasing condition, such abrupt changes in sweetness were absent, likely due to the mitigated habituation, resulting in an increased AUC. Additionally, the proportion of participants reporting a decrease in sweetness impressions was reduced by about half, supporting the effectiveness of the increasing condition in alleviating habituation.

In the non-habituated group, no sharp decrease in sweetness was observed under the constant condition, unlike in the habituated group. Furthermore, the proportion of participants reporting a decrease in sweetness under the constant condition was lower than in the habituated group. This suggests that the contrast effect did not occur, leading to a state less prone to habituation. Consequently, no mitigating effect on habituation was observed under the increasing condition, resulting in no significant difference in AUC.

Regarding the AUC under the decreasing condition, no significant differences were observed between the decreasing and constant conditions in either the habituated or non-habituated groups. These results refuted both H3 [Dec. <Const. (habituated)] and H4 (Dec. > Const. (non-habituated)).

In the habituated group, sweetness remained slightly higher than in the constant condition up to 12 s. This suggests the potential contribution of perceptual constancy due to the initially strong sweetness, similar to the high-zero-low salt concentration variation method described by Dijksterhuis et al. (2014). However, a sharp drop in sweetness was observed at 15 s. At this time point, the combination of sweetness reduction due to both the decrease in sugar concentration and habituation may have amplified the reduction through a contrast effect.

In the non-habituated group, sweetness under the decreasing condition was slightly lower than in the constant condition throughout the consumption process. This group exhibited a sensitization characteristic under the constant condition, where sweetness increased up to 6 s. However, under the decreasing condition, the reduction in sugar concentration likely prevented such sensitization, resulting in slightly lower sweetness throughout. Despite the sugar concentration decreasing to ~67% from the start to the end of the trial, the perceived sweetness reduction was limited to about 93%, suggesting that perceptual constancy effects may have played a role.

In the increasing condition, a sweetness enhancement effect was observed in the habituation group. In contrast, no sweetness enhancement effect was observed in either group under the decreasing condition. These findings suggest that in the design of gradual sugar content changes, an increasing pattern, rather than a decreasing one, is more likely to enhance sweetness through the alleviation of habituation.

Different results were observed between the habituation and non-habituated groups under the increasing condition. This difference may be influenced by gender-based differences in sweetness sensitivity. The gender composition differed between the groups: the habituated group included a higher proportion of male participants, whereas the non-habituated group included more females (Figure 12B). Males are generally considered to have lower sweetness sensitivity compared to females (Melis et al., 2022). Additionally, repeated stimuli of lower intensity are more likely to induce habituation (Rankin et al., 2009). In the habituated group, the higher proportion of males may have resulted in overall lower perceived sweetness intensity, creating a condition more conducive to habituation. In contrast, the higher proportion of females in the non-habituated group likely led to higher overall perceived sweetness intensity, making habituation less likely. These differences in sweetness sensitivity are thought to be influenced by innate factors such as taste bud density and modified by dietary habits in daily life.

The use of perceptual constancy bias through high-zero-low changes in salt concentration (Dijksterhuis et al., 2014) and the reduction of the contrast effect via habituation alleviation proposed in this study are both methods that enhance taste by leveraging biases in temporal perception of stimuli. This paragraph discusses the applicability of each approach. In prior research, the high-zero-low method demonstrated a saltiness enhancement effect through a pattern of high-zero-low concentration (e.g., 70%–0%–30%). However, in this study, a simple decreasing condition, where the initial sugar concentration was high and gradually decreased, did not result in enhanced sweetness. This difference in effectiveness suggests that utilizing perceptual constancy bias requires increasing the taste stimulus at the end to improve the post-estimation value of the diminished central taste, rather than just starting from a high concentration. Conversely, in this study, setting the initial sugar concentration to 0.8 times the baseline and gradually increasing it showed a sweetness enhancement effect for the habituated group. This indicates that when habituation lowers the perceived taste at the end, reducing the contrast effect is more effective than utilizing perceptual constancy for enhancing sweetness.

This study suggested that a gradual increase in sugar concentration mitigates habituation and enhances perceived sweetness. In the pulse taste stimulation method using sucrose solutions by Burseg et al., the maximum increase in sweetness evaluation compared to constant presentation was 14% (Burseg et al., 2010). In comparison, the gradual sugar concentration increase method proposed in this study showed a 15% increase in sweetness evaluation compared to constant presentation. Although direct comparisons are not possible due to differences in evaluation methods, this approach appears to provide effects comparable to pulse taste stimulation.

In the method proposed by Dijksterhuis et al., which involved high-zero-low changes in salt concentration, the proportion of participants reporting enhanced sweetness compared to the constant condition reached a maximum of 62%. In contrast, the gradual sugar concentration increase method in this study resulted in 90% of participants in the habituated group reporting enhanced sweetness. Although direct comparisons are difficult due to differences in taste modality, evaluation methods, and participant categorization, these results suggest that our method could be effective specifically for individuals who show habituation tendencies during continuous consumption.

Furthermore, the gradual sugar concentration variation method proposed in this study facilitates the mitigation of habituation, which is challenging for pulse taste stimulation and high-zero-low variation methods, enabling sustained sweetness perception throughout the eating process. This method might minimize the conscious perception of taste changes compared to pulse stimulation or high-zero-low variations. This characteristic could be advantageous when considering practical applications in eating experience design, though further investigation in real eating scenarios would be needed.

This study contributes to our understanding of habituation mechanisms. While previous research has elucidated the mechanisms of habituation (Kandel et al., 2021) and its characteristics (Rankin et al., 2009), providing insights into the effects of stimulus intensity and frequency on habituation and methods for dishabituation, the impact of gradually changing stimulus intensity on habituation had not been clarified. Our findings suggest that gradual increases in stimulus intensity may help mitigate habituation, at least in the context of sweetness perception during short-term consumption.

Furthermore, the method proposed in this study demonstrates the potential to enhance sweetness perception without altering the total sugar intake or introducing additional taste components. While further research is needed to validate these effects in various food products and longer consumption periods, this approach might contribute to the development of strategies for sugar reduction while maintaining taste satisfaction.

## 7 Limitation and future works

This study has several limitations. First, it did not evaluate post-consumption taste intensity estimates. These estimates are important as they reflect the taste of the food retained in memory, which is closely related to the perceived value of the food. Particularly, the beginning and end of a food experience are likely to be weighted more heavily in these estimates compared to the middle (Dijksterhuis et al., 2014). While the real-time sweetness evaluations conducted after each swallow in this study were appropriate for demonstrating the proposed method's effect on mitigating habituation, the total sweetness evaluation–calculated as the sum of five swallows without weighting–does not necessarily align with the taste retained in memory after consumption. Future studies should incorporate experimental designs that allow for the evaluation of post-consumption taste intensity estimates.

Second, the application time in this study was limited to 15 s, and the applicability of the method to longer durations remains unclear. Since adaptation processes can occur over several minutes (Schiffman et al., 1994), extending the consumption time might reveal more pronounced effects.

Third, the study did not explore the applicability of the method to solid foods. In methods such as pulse taste stimulation and high-zero-low salt concentration variations, differences in concentration within bread or cream have enabled their application to solid foods (Noort et al., 2010; Dijksterhuis et al., 2014). Similarly, this method could potentially be implemented by introducing sugar concentration gradients within food. For instance, in the case of jelly, it may be feasible to produce a multilayered structure with different sugar concentrations. As the consumer eats from the top, the gradual transition in sweetness could be achieved through the vertical layering of the product. Future research should investigate its application to solid sweet foods.

Finally, the study did not examine the potential for maximizing sweetness by combining the proposed method with existing approaches. Several techniques for enhancing sweetness, including intense sweeteners, chemical additives, and pulse taste stimulation, have been proposed. Future research should explore the combined effects of these methods with the gradual sugar concentration increase method presented in this study.

## 8 Conclusion

This study proposed a method to enhance perceived sweetness without altering total sugar intake by gradually increasing sugar concentration during the eating process. The hypothesis was that this method could mitigate sweetness habituation and enhance perceived sweetness.

The results showed that in groups prone to habituation to consistent sweetness stimuli, the proposed method enhanced perceived sweetness by an average of 15%, while no enhancement was observed in groups less susceptible to habituation.

These findings suggest that a gradual increase in sugar concentration during the eating process can effectively enhance perceived sweetness in individuals prone to habituation to consistent sweetness stimuli.

This method contributes to the understanding of habituation mechanisms and offers a potential practical approach for enhancing cumulative perceived sweetness in food experiences where habituation occurs, while maintaining sugar intake at lower levels. Through this approach, we hope to contribute to the development of strategies that could help promote taste satisfaction without increasing sugar consumption, potentially supporting efforts in obesity and type 2 diabetes prevention.

## Data Availability

The original contributions presented in the study are included in the article/supplementary material, further inquiries can be directed to the corresponding author.

## Footnotes

^1^https://www.youtube.com/watch?v=UbmrZQWoU_8 (accessed: 2024-01-14).
